# Using Diagnostic Radioentomology for Non-Invasive Observations of Colonies of the Bumblebee, *Bombus terrestris*

**DOI:** 10.1673/031.012.8501

**Published:** 2012-07-20

**Authors:** Mark K. Greco, Ben M. Sadd

**Affiliations:** ^1^ INVERT Centre, Department of Electrical and Electronic Engineering, University of Bath, Bath BA2 7AY, UK; ^2^ Department of Biology and Biochemistry, University of Bath, BA2 7AY United Kingdom; ^3^ Experimental Ecology, Universitätstrasse 16, ETH-Zentrum CHN J12.2, CH-8092, Zürich, Switzerland

## Abstract

Bumblebees have been the focus of a broad range of scientific research due to their behavior, social life, and a number of other intriguing traits. Current methods for examining their nest structure, such as natal cells and contents of storage cells, are destructive in nature because the cells need to be opened for physical inspections. This research describes how the internal structures of the artificial nests of the bumblebee *Bombus terrestris* L. (Hymentoptera: Apidae) were non-invasively viewed and assessed by using diagnostic radioentomology. For the first time, *B. terrestris* nest structures, and their contents such as larvae, pupae and eggs, were non-invaseively viewed and assessed. This technique will enable future experiments to take morphological measurements of egg, larval, and pupal development over time. Moreover, combining these measurements with measures of food-storage will provide a good assessment of colony health. The method will also allow tracking of individually marked adults, to monitor their behaviour and help gain a better understanding of the processes involved in the global declines of *B. terrestris*, which will in turn promote better management of these valuable pollinators.

## Introduction

There are approximately 400 species of bumblebees *Bombus* spp. L. (Hymenoptera: Apidae) (Heinrich 1979). They are large, pilous, long-tongued, social bees that live in colonies within soil or log cavities in the temperate regions of the globe (Michener 1974; Heinrich 1979; Delaplane and Mayer 2000). Colony health of these bees, which will ultimately determine the population health, and thus stability of the pollinator communities, is of upmost importance.

Bumblebees have been the focus of a broad range of scientific research due to their behavior, social life, and a number of other intriguing traits. For example, bumblebees have been utilised in research on social evolution (Page and Metcalf 1982; Pamilo 1991), development (Hartfelder et al. 2000; Yerushalmi et al. 2006), plant-pollinator interactions (Gegear and Burns 2007; Suzuki et al. 2007; Cooley et al. 2008)), learning (Leadbeater and Chittka 2007; Ings et al. 2009), invasion biology (Schmid-Hempel et al. 2007), host-parasite ecology (Durrer and Schmidhempel 1994; Baer and Schmid-Hempel 2003; Schmid-Hempel and Reber Funk 2004; Otti and Schmid-Hempel 2007), and community ecology (Hatfield and LeBuhn 2007; Williams et al. 2007). Furthermore, bumblebees are key pollinators in both natural and agricultural settings, and in many cases can be more efficient and efficacious than honeybees in this regard. For instance, Lucerne (alfalfa: *Medicago sativa* L.) and red clover (*Trifolium pratense* L.; Fabales: Fabaceae) flowers hide their pollen and stigmas within a keel, which has to be removed for pollination. Bumblebees sonicate while foraging, and are thus able to access this pollen, thereby pollinating as they forage.

Although honeybees produce internidal buzz frequencies during dance communication and heating behaviour, they do not sonicate while foraging on flowers as bumblebees do (Westerkamp and Gottsberger 2000). Buzzing is necessary to release pollen from poricidal anthers on flowers such as of tomato, as previously discussed. Farmers seeking pollinators for greenhouse tomatoes have used bumble bees successfully since a solution to “artificial” queen hibernation was developed (Roseler 1985; Velthuis 2002), enabling colonies to be produced all year.

The significance of bumblebees comes into focus given the threat that pollinators currently face worldwide. This crisis to pollinators has been epitomised by the recent honeybee colony collapse disorder (Cox-Foster and Vanengelsdorp 2009), but the bumblebee has also suffered population declines in many areas over a number of decades (Biesmeijer et al. 2006; Fitzpatrick et al. 2007; Grixti et al. 2009). Indeed, seed production from lucerne and red clover has decreased in conjunction with a reduction of bumblebee populations globally. Conversely, seeds from lucerne and red clover are produced in New Zealand, where bumblebees have been introduced from Europe, and are currently not in decline. There is now enough seed produced in New Zealand to export back to Europe (Osborne et al. 1991).

As outlined above, information about bumblebee colony health is important for addressing a number of both basic scientific and applied issues. In this study, the ability to monitor bumblebee colony status non-invasively using DR (Diagnostic radioentomology) was assessed, and the potential for providing information on egg, larval, pupal, and adult populations, was shown.

## Materials and Methods

DR was performed on two managed artificial nests of *B. terrestris* at the end of an unrelated experiment in October 2009, using methods described in Greco et al. 2005, using a Philips Brilliance CT 16-slice scanner (Philips Healthcare, 5680 DA Best, The Netherlands). To ensure that there were no biological effects from the radiation, scan times were limited to 30 seconds, which produced an average dosage of 7.9 mGy per nest (Kanao et al. 2003; Greco et al. 2005; Greco et al. 2006). The nest material was made from a combination of gypsum, polystyrene, and cement. Colonies were analysed using BeeView 3D visualisation software (Disect Systems Ltd, Suffolk, United Kingdom). 3D reconstructions were performed to better visualise the spatial relationships between the nest structures. 2D reconstructions in coronal, sagittal, and axial views were performed to improve image resolution. The colonies used were small because they were subjected to persistent removal of workers to simulate natural mortality rates. Colonies were kept under red-light illumination at 27 ± 1° C with sugar water (Apilnvert®) and pollen provided *ad libitum*.

## Results

**Figure 1.**
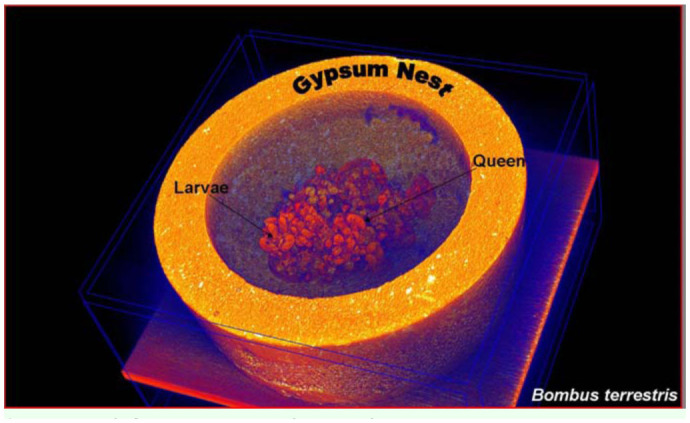
A 3D DR image of an artificial nest containing a *Bombus terrestris* colony. The queen and one of the larvae (arrows) were observed on the upper surface of the nest during the scan. High quality figures are available online.

**Figure 2.**
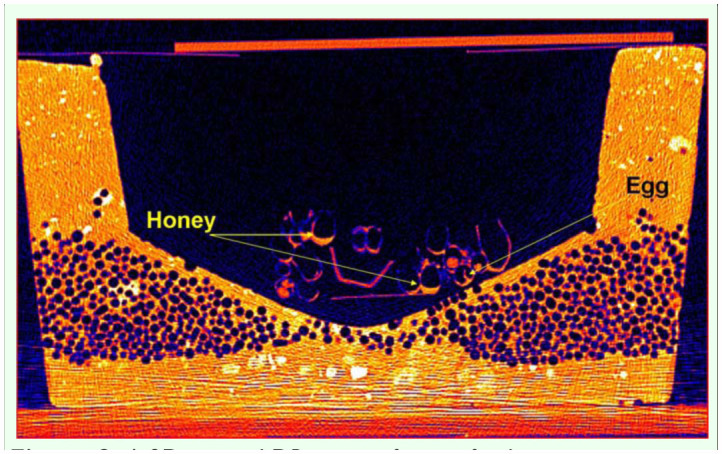
A 2D coronal DR image of an artificial nest containing a *Bombus terrestris* colony. The storage pots and natal cells were observed in cross-sectional detail including evidence of minimal storage of sugar water (honey), and a developing egg (arrows), indicating that oviposition was occurring in the colony. High quality figures are available online.

**Figure 3.**
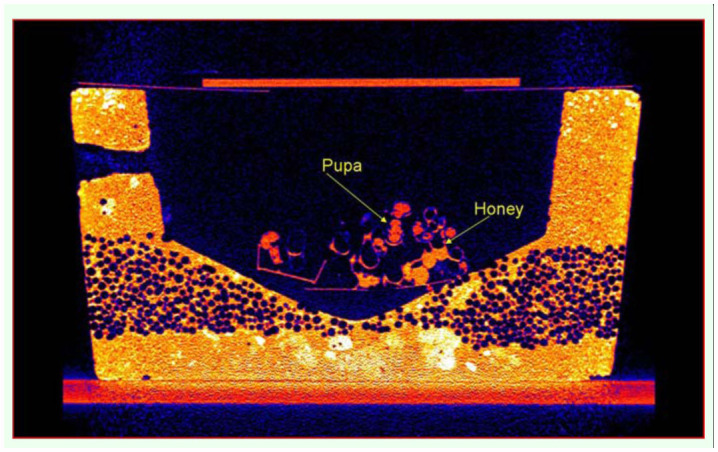
A 2D sagittal DR image of an artificial nest containing a *Bombus terrestris* colony. The storage pots and natal cells were observed in cross-sectional detail including evidence of minimal storage of sugar water (honey), and a developing pupa (arrows). Details of the head, thorax, abdomen, and the bases of two legs of the pupa were observed. High quality figures are available online.

**Figure 4.**
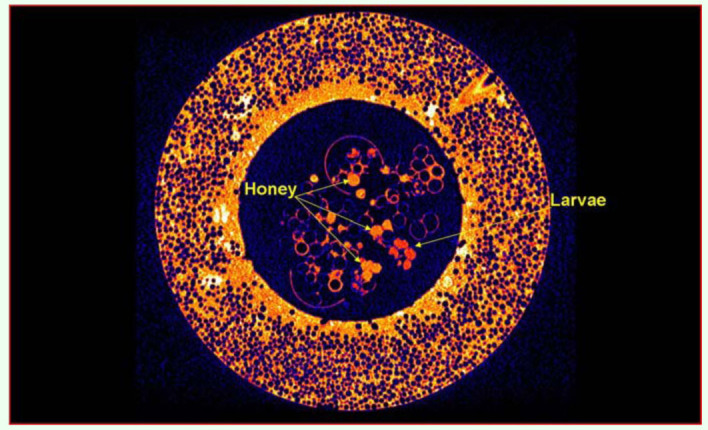
A 2D axial DR image of an artificial nest containing a *Bombus terrestris* colony. The storage pots and natal cells were observed in cross-sectional detail including evidence of sugar water storage (honey), and four developing larvae (arrows). The axial views enable observations of the entire nest showing evidence that most cells were empty. High quality figures are available online.

Eggs, larvae, pupae, adult workers, and the Queen were easily identified. Figure 1 shows the 3D detail that can be achieved using DR to visualize individuals and structures in a managed bumblebee colony. The coronal 2D views showed cross-sections of storage pots containing minimal quantities of stored sugar water, and evidence of ovipositing behavior in the form of new eggs (Figure 2). Given that these colonies were late in the colony cycle, it was not possible to determine if these were queen or worker produced eggs. The sagittal 2D views (Figure 3) showed details of the head, thorax, abdomen, and two legs of a pupa. The axial 2D views (Figure 4) enabled observations of the entire nest, including evidence that most cells were empty, and that there was generalized separation of clumps of larval brood and stored sugar water.

## Discussion

DR allowed the internal structures of the artificial nests of *B. terrestris* to be viewed and assessed non-invasively. Current methods for examining natal cell and sealed storage pot contents are destructive in nature because the pots and cells need to be opened for physical inspections. Furthermore, the multi-layered structure of a bumblebee nest, as can be observed in Figures 2 and 3, makes any uniform physical sampling of cell contents difficult. In this experiment, DR enabled accurate non-invasive assessment of *B. terrestris* nest structures and their contents to be made for the first time. In addition, non-invasive egg identification and assessment was made possible. The eggs were not measured in this experiment; however, in future experiments, eggs can be localized, identified (by their DR x, y & z spatial coordinates), and measured, and their subsequent development can be tracked over time. These features of DR will enable a suite of behavioral and colony health experiments to be performed on *B. terrestris*. For example, labelled sugar water can be introduced to the colony, and sugar water storage pattern behavior can be observed non-destructively, without modifying foraging and storage behavior. Also, colonies can be infected with known pathogens so that individual egg/larval/pupal development can be monitored non-invasively to observe the temporal effects of those pathogens. Pathogens have been implicated in recent bumblebee declines (Cameron et al. 2011), and fine scale temporal assessment of a number of colony traits will aid the establishment of causal links between pathogens and bumblebee health.

A limitation of DR is bee movements, which cause motion artefacts; however, as new motion error algorithms develop, this limitation will be addressed. In combination with well-designed experiments, DR has the potential to unearth noteworthy aspects of bumblebee biology, and impacts of important environmental variables. For instance, the knowledge acquired from these DR experiments could be used to gain a better understanding of the processes involved in the global declines of bumblebees, which will in turn enable better management of these valuable pollinators.

## Abbreviation

DR,: diagnostic radioentomology
